# The effectiveness and characteristics of pregnancy yoga interventions: a systematic review protocol

**DOI:** 10.12688/hrbopenres.12967.2

**Published:** 2020-01-08

**Authors:** Lisa Corrigan, Jessica Eustace-Cook, Patrick Moran, Deirdre Daly

**Affiliations:** 1 School of Nursing & Midwifery, University of Dublin, Dublin, Ireland; 2 Library of Trinity College Dublin, University of Dublin, Dublin, Ireland

**Keywords:** Pregnancy, Yoga, Systematic review, Intervention

## Abstract

**Background: **The purpose of this review is to systematically examine the reported clinical effectiveness of pregnancy yoga.  The review will use the FITT (frequency, intensity, time/duration and type) principle of physical activity to characterise the different types of yoga interventions that have been evaluated in the included studies.  Studies will be categorised as effectiveness or efficacy studies and this continuum of efficacy versus effectiveness will be incorporated into the full review.

**Methods/design: **The following electronic databases will be searched using a detailed search strategy: MEDLINE, PsycINFO, EMBASE, CINAHL, WHOLiS, AMED, ScieLo, ASSIA and Web of Science. Randomised control trials and quasi-experimental studies examining pregnancy yoga and reporting on effect will be included. Titles, abstracts and full articles will be screened by two investigators independently to identify eligible studies.  The Cochrane Collaboration’s Risk of Bias Assessment tool will be used to assess study quality.  Quality of the evidence will be evaluated using the GRADE criteria. A standardised data extraction form will be used to extract data. Effect sizes will be estimated using mean differences for continuous outcomes, and relative risks for dichotomous outcome. Where possible, pooling of effect estimates will be done using a random effect model.  The outcomes of interest are quality of life, stress, anxiety, depression, mode of birth, labour duration and pain management in labour.

**Discussion: **This review will synthesise the best available evidence on the effectiveness of yoga during pregnancy and provide valuable high-quality information for clinicians and health policymakers. Findings will be disseminated through publication in a peer-reviewed journal and presentation at relevant conference proceedings. The review will make recommendations for the appropriate volume, intensity and type of pregnancy yoga for maximum effect and may have implications for policy and practice relating to pregnancy yoga as an intervention.

**Registration:** PROSPERO, CRD42019119916.  Registered on 11th January 2019.

## Abbreviations

FITT – frequency, intensity, time/duration and type; GRADE – Grades of recommendation, assessment, development and evaluation; PRISMA-P – Preferred reporting items for systematic reviews and meta-analysis protocols; QoL – Quality of life; RCT – Randomised control trial

## Introduction

Pregnancy is a time of significant change which can elevate stress levels and increase responsivity to external stressors ^1, 2^. These external stressors can lead to adverse perinatal outcomes such as perinatal depression, pregnancy induced hypertension and pre-eclampsia with prolonged maternal stress associated with both preterm birth and low birth weight ^3– 5^. Excessive maternal stress during pregnancy has also been linked to children’s later attention deficit hyperactivity disorder symptomatology as well as lower executive functioning ^6, 7^. Adverse levels of stress during pregnancy are therefore a cause for concern and represent a significant public health concern. In recent years yoga has been adapted for the pregnant body and is a common complementary and alternative medicine used by pregnant women ^8^. It is a low impact exercise that can easily be adapted to suit individual needs. A review of yoga’s effect on stress, reported in 25 out of 35 included trials with 1398 participants that a yoga intervention significantly reduced stress ^9^. The same review also identified limitations in yoga trials reviewed, including small sample sizes, lack of randomisation and not using control groups.

The word yoga, meaning ‘union’, is a mind-body-spirit practice that can include meditation, breath awareness, Asanas or postures and relaxation. It is thought to alter nervous system regulation, physiology, psychological well-being and physical fitness. While no pooled international data exists on usage among pregnant women, an Australian study by Steel *et al.* ^10^ showed that between 17% and 19% of Australian women practice pregnancy yoga. Curtis *et al.* ^2^ in a systematic review of yoga for pregnant women with six studies and 689 participants concluded that overall the evidence in support of pregnancy yoga was positive with randomised control trials (RCTs) indicating improvements in stress levels, quality of life (QoL), autonomic nervous system functioning and labour parameters, such as comfort, pain and duration. However, they also stated that due to the limited number of RCTs and absence of double blind RCTs, they included studies that were randomised but not double-blinded and noted that controlled trials that lacked randomisation. Overall, methodological issues with the published literature and insufficient published trials included in the review made it impossible to report definitive conclusions. A further systematic review of the effects of pregnancy yoga on birth outcomes identified eleven studies all demonstrating the benefits of a pregnancy yoga intervention with no adverse effects reported ^11^. The included studies varied considerably in length and intensity of the yoga intervention, the degree of supervision of the intervention, measurement tools utilised, sample population and outcomes measured. This suggests that the effects of pregnancy yoga may not necessarily be dependent upon the length and intensity of the practice, therefore factors contributing to effectiveness as reported in the literature require further exploration.

While the evidence in support of pregnancy yoga is accumulating, systematic reviews of the literature identify a need for more robust studies using transparent, reproducible yoga interventions with defined outcomes to accurately capture effectiveness. Previous reviews have not synthesised data across studies to calculate effectiveness for a range of reported outcomes or the characteristics of the pregnancy yoga intervention being provided. The objective of this systematic review is to examine the published evidence on pregnancy yoga, describe the characteristics of each intervention using the frequency, intensity, time/duration and type (FITT) principle of physical activity and through meta-analysis, assess the overall effects of pregnancy yoga on a range of identified outcomes ^12^. It will also critically review the methodological quality of the studies identified to guide future research in the field. Investigation of the effectiveness of pregnancy yoga for improved pregnancy and birth outcomes is a topical area that has potential to alter practices and antenatal care. Understanding the factors that contribute to effective pregnancy yoga interventions can help guide the development of pregnancy yoga programmes that optimise effect and also ensure safety for both mother and baby.

## Methods/design

This protocol has been developed according to the PRISMA guidelines for systematic review protocols (PRISMA-P) ^13^. A complete PRISMA-P checklist for this protocol is provided in *Extended data:* PRISMA-P Checklist. The protocol has been registered on PROSPERO, registration number CRD42019119916.

### Aim

The aim of this systematic review is twofold:

- To assess the effects of the pregnancy yoga interventions used in RCTs and quasi-experimental studies on stress as measured by salivary cortisol and/or self-report questionnaires, QoL, anxiety, depression, mode of birth, labour duration and pain management in labour.
- To identify the frequency (how often?), intensity (how much/dose?), timing (how long?) and type (what type?) characteristics of pregnancy yoga interventions used in RCTs and quasi-experimental studies.

### Eligibility criteria

The following criteria will be used to select studies for inclusion in the systematic review:

1. *Study design:* Any primary study that investigates a pregnancy yoga intervention within a RCT or quasi-experimental study with a control before and after design will be considered for inclusion as these study designs constitute the most robust form of clinical evidence. Case control studies, crossover trials and cross-sectional studies will be excluded.
2. *Participants:* Included studies will involve pregnant women of any gestation.
3. *Interventions:* Studies where yoga is the primary intervention delivered to a sample of pregnant women will be considered for inclusion. Multimodal interventions which deliver yoga in conjunction with other treatments for pregnant women will be excluded.
4. *Comparators:* Studies with pregnant women of any gestation receiving usual care or any active treatment other than yoga.
5. *Outcomes:* The outcomes will be QoL reported by standardised measures, birth outcomes (labour duration, pain management in labour and mode of birth), stress documented by both self-reported questionnaires and physiological measurements, anxiety and depression as measured by standardised questionnaires and/or clinical diagnosis. The different treatment outcomes will be collated and described as their measurement may vary in methods and units.
6. *Setting:* RCTs and quasi-experimental studies conducted in all settings (hospital, community, health centre) and all geographical locations will be included. There will be no restriction by type of setting.
7. *Language:* Only studies available in English will be included.

### Information sources

A systematic literature search will be performed using the following databases: (EBSCO)Medline (1946-), CINAHL (1981-), PsycINFO (1990-), (Ovid)Embase (1966-), AMED (1985-), WHOLiS, Web of Science (1864-), ScieLo (2002-) and ASSIA (1970-). Reference lists from included studies and relevant reviews will be screened to ensure coverage of the literature. In addition, Google Scholar results for the last year of publication, will also be visually scanned for any additional material. No restrictions on date of study publication will be included in the search, and all articles available in English will be considered. The review will only include peer-reviewed published studies.

### Search strategy

The search strategy was developed by the primary author (LC) and supported by a subject librarian with systematic review expertise (JEC), with input from all authors. Citation and bibliographic searches will be conducted on all included studies to identify additional relevant studies for inclusion. The search will be updated and rerun just before final analysis is conducted and further studies retrieved for inclusion. *Extended data:* Search Strategy contains sample search terms and a search strategy example. Conference proceedings, editorials, commentaries, and book chapters/book reviews will be excluded.

### Study records

Literature search results will be exported to EndNote X9 and duplicate records deleted using the ‘remove duplicates’ function and by manually screening results for accuracy (LC and JEC) ^14^. Titles and abstracts of identified articles will be imported to Covidence (JEC), a web-based software platform designed to support citation screening and collaboration among multiple authors ^15^.

### Selection process

Two reviewers (LC and DD) will independently screen all titles and abstracts of retrieved studies. Articles clearly not meeting the established inclusion/exclusion criteria will be excluded. If there is disagreement about inclusion, the abstract will be reviewed by a third author (PM) to determine suitability. Following this, two reviewers will independently screen the full text articles of abstracts identified to select the studies to be included (LC and DD). The reference lists of included studies will be scanned to identify any relevant additional studies. Both reviewers will independently screen the full text of these additional articles to determine inclusion/exclusion (LC and DD). Third-party arbitration (PM) will be available to resolve any inconsistencies in the selection of studies for inclusion/exclusion.

If necessary, study authors will be contacted up to three times to resolve questions about eligibility (LC). Reasons will be recorded for excluding studies. Studies will only be included once for evaluation purposes but multiple publications from the same study will be reported. A Preferred Reporting Items for Systematic Review and Meta-Analysis (PRISMA) flow diagram will show the overall process of study selection and summarise the inclusion and exclusion of studies at each stage of the review.

### Data collection process

A standardised data extraction tool will be developed specifically for this review based on recommendations provided in the Cochrane Handbook of Systematic Reviews of Interventions (LC) ^16^. Data will be extracted on study design and methods, sociodemographic characteristics, inclusion and exclusion criteria, study setting, details of experimental intervention and comparison intervention, duration of follow-up and outcomes studied, and extent of effectiveness. Two authors (LC and PM) will independently extract data using the standardised form, piloted in advance to ensure accuracy. Any differences of opinion between the two reviewers will be resolved by a third review author (DD) and consensus achieved. If necessary, study authors will be contacted up to three times to provide further details. Data will be entered into Review Manager Software and checked for accuracy ^17^.

### Outcomes

All outcomes collected at every time point will be included in the review. The primary outcomes of interest will be stress, anxiety, depression and QoL. Secondary outcomes will be birth outcomes including labour duration, pain management in labour and mode of birth. All reported outcome measures will be documented.

### Risk of bias

Risk of bias of included studies will be assessed using the Cochrane Risk of Bias Assessment tool that contains several items under 7 categories, such as random sequence generation, allocation concealment, blinding of participants and investigators, the blindness of outcome assessments, incomplete outcome data, selective outcome reporting, and other biases ^16^. Based on the assessment, the studies will be evaluated as low, unclear, or high bias.

Two reviewers will independently assess each article for risk of bias (LC and PM). Discrepancies will be resolved by discussion or if required referral to a third reviewer (DD). Where reported information is unclear or where data are missing all attempts will be made to contact the primary authors for clarification.

### Assessment of the body of evidence – the GRADE approach

Quality of the evidence will be evaluated using the Grades of Recommendation, Assessment, Development and Evaluation (GRADE) approach ^18^. This will assess the quality of evidence for specific outcomes. The evidence can be downgraded from ‘high quality’ by one level for serious or two levels for very serious limitations, depending on assessments of risk of bias, indirectness of evidence, serious inconsistency, imprecision of effect estimates or publication bias. Two reviewers will independently assess each article for quality (LC and PM). Discrepancies will be resolved by discussion or if required referral to a third reviewer (DD). Where reported information is unclear or where data are missing all attempts will be made to contact the primary authors for clarification.

GRADE profiler will be used to import data from Review Manager Software version 5.3 (RevMan 5.3) in order to create the summary of findings table (LC) ^17, 19^. A summary of intervention effect and a measure of quality according to the GRADE approach will be presented for the following outcomes of interest:

- Maternal stress
- Maternal QoL
- Maternal anxiety
- Maternal depression
- Labour, pain management and mode of birth outcomes as reported in the studies

### Data synthesis

Two review authors (LC and PM) will extract the following study characteristics from included studies:

1. *Methodological/trial design:* study design, method of randomisation, method of allocation concealment, total duration of study, details of any ’run-in’ period, study setting, withdrawals, duration of treatment period, duration of follow-up, information on sponsorship/funding.
2. *Participants:* number of participants allocated to each treatment group, mean age/age range, inclusion and exclusion criteria.
3. *Interventions:* total number of intervention groups with number of participants in each group, type, duration and frequency of intervention, presence of supervision, comparison.
4. *Outcomes:* number of participants with each outcome per group will be noted. Primary and secondary outcomes will be specified and collected, and time points will be reported. Two review authors (LC and PM) will independently extract outcome data from included studies. We will resolve disagreements by consensus or by involving a third person (DD). One review author (LC) will transfer data into the RevMan 5.3 file ^17^.

Heterogeneity between studies will be assessed using RevMan 5.3 by comparing study settings, populations and study design, using the I ^2^ statistic ^16^. Results from included studies will be presented as summary risk ratios with 95% confidence intervals for dichotomous outcomes. The mean difference will be used for continuous data where outcomes are measured in the same way between trials. Where trials measure the same outcome using different methods the standardised mean difference will be used. We will combine the outcome measures from the individual trials through meta-analysis where possible (clinical comparability of populations, interventions, outcomes and time of assessment between trials) using a random-effects model, because we expect some between-study variation. Should the data be considered not sufficiently similar to be combined in a meta-analysis, the results from clinically comparable trials will be described qualitatively in the text. Statistical heterogeneity will be assessed in each meta-analysis using the T ^2^, I ^2^ and chi square statistics ^16^. For included studies, levels of attrition will be recorded and the impact of including studies with high levels of missing data on assessment of treatment effect will be explored further using sensitivity analysis.

The information will be presented in narrative and tabular format to summarise the characteristics and results of the included studies and explain the average treatment effect and outcomes within and between studies.

### Sensitivity analysis

For the primary and secondary outcomes, we will compare analyses including and excluding trials at high risk of bias (as defined in assessment of risk of bias in included studies) in order to explore the impact of risk of bias on estimates of treatment effects.

### Analysis of subgroups or subsets

Subgroup analysis will be conducted where appropriate to stratify results by:

(i) Frequency (How often?)
(ii) Intensity (Dose/How many?)
(iii) Timing of yoga (What is the duration of each session?) and
(iv) Type (Which style of yoga?)

Separate comparisons and analyses for primary and secondary outcomes will be performed where possible and when appropriate.

### Dissemination of results

The systematic review will be disseminated in peer-reviewed journals. The dataset generated and/or analysed during the study shall be available from the corresponding author on reasonable request.

### Amendments

Any amendments to this protocol will be described in a table including the date of each amendment as well as a description of and rationale for this. The PROSPERO register will remain updated with the protocol and any amendments made.

### Ethics approval and consent to participate

Ethical approval is not required for this study as it will not involve conducting experimental research, nor include identifying personal data.

## Study status

Database searching and formal screening of search results against eligibility criteria are complete.

## Discussion

Pregnancy yoga can be adjusted to suit the pregnant body and is a common complementary and alternative medicine used by pregnant women ^8^. While the body of evidence supporting its positive impact on pregnancy and birth outcomes is growing there is a need to pool evidence from trials to accurately measure treatment effect and the factors that contribute to these reported benefits ^2, 11^.

This will be the first systematic review of pregnancy yoga to incorporate the FITT principle of physical activity into the analyses ^12^. Thus far, there is limited evidence identifying the optimal mode, frequency, intensity and duration of pregnancy yoga that should be recommended for maximum benefit. Mottola and Artal ^20^ propose following the FITT principle as guidance for women who are exercising during pregnancy and highlight that these factors may change as a pregnancy progresses. By exploring these factors, the findings from this review may assist maternity care providers and exercise practitioners in deciding to recommend or prescribe pregnancy yoga as an intervention to support better pregnancy and birth outcomes.

## Data availability

### Underlying data

No data is associated with this article.

### Extended data

Open Science Framework: The effectiveness and characteristics of pregnancy yoga interventions: a systematic review protocol, https://doi.org/10.17605/OSF.IO/J3X4M ^21^

This project contains the following extended data:

- Supplementary File 1. Sample Search Strategy

### Reporting guidelines

Open Science Framework: PRISMA-P checklist for ‘The effectiveness and characteristics of pregnancy yoga interventions: a systematic review protocol’, https://doi.org/10.17605/OSF.IO/J3X4M ^21^

Data are available under the terms of the Creative Commons Zero “No rights reserved” data waiver (CC0 1.0 Public domain dedication).
