# Supplementary material for: The effectiveness and characteristics of pregnancy yoga interventions: a systematic review protocol
Source: HRB Open Res. 2020 Jan 8;2:33. Originally published 2019 Nov 20. [Version 2] doi: 10.12688/hrbopenres.12967.2 (PMC7140767; doi:10.12688/hrbopenres.12967.2)
Supplement: Peer review history

# Peer review: Reviewer response for version 2

**DOI:** 10.21956/hrbopenres.14077.r27060

**Published:** 2020-02-27

**Authors:** Patricia Kinser

**Affiliations:** 1 School of Nursing, Virginia Commonwealth University, Richmond, VA, USA

I confirm that I have read this submission and believe that I have an appropriate level of expertise to confirm that it is of an acceptable scientific standard.

# Peer review: Reviewer response for version 2

**DOI:** 10.21956/hrbopenres.14077.r27061

**Published:** 2020-01-08

**Authors:** James Newham

**Affiliations:** 1 Department of Psychology, Northumbria University, Newcastle upon Tyne, UK

No additional comments.

I confirm that I have read this submission and believe that I have an appropriate level of expertise to confirm that it is of an acceptable scientific standard.

# Peer review: Reviewer response for version 1

**DOI:** 10.21956/hrbopenres.14052.r26987

**Published:** 2019-12-17

**Authors:** James Newham

**Affiliations:** 1 Department of Psychology, Northumbria University, Newcastle upon Tyne, UK

While there have been previous systematic reviews examining the effects of yoga on health outcomes, the current authors take a novel approach by trying to standardise the content of yoga interventions.

 Due to the nature of yoga as an established intervention, it is difficult to run it as an intervention where participants are blinded. Therefore many studies will by default be considered low quality by how most quality appraisal criterion value blinding with high quality. This makes it difficult to determine whether differences between studies is due differences in the quality of the study.

 As an alternative, yoga interventions have typically been heterogenous in intensity and format, and this may explain previous differences between studies in their findings. Using the FITT approach as a method to standardise interventions for sensitivity analyses, the authors may be able to explain what format a yoga intervention needs to take to be most effective.

I confirm that I have read this submission and believe that I have an appropriate level of expertise to confirm that it is of an acceptable scientific standard.

# Response

**Published:** 2020-01-07

**Authors:** Lisa Corrigan

**Affiliations:** Trinity College Dublin, Ireland

Dear James

Thank you for taking the time to review our protocol it is much appreciated.  We are delighted that you appear to share our interest in exploring the FITT principle of physical activity as it applies to the yoga practices within identified studies.  We hope it will bring added understanding to the practice of yoga in pregnancy regarding type, dose, and duration.

# Peer review: Reviewer response for version 1

**DOI:** 10.21956/hrbopenres.14052.r26949

**Published:** 2019-11-27

**Authors:** Patricia Kinser

**Affiliations:** 1 School of Nursing, Virginia Commonwealth University, Richmond, VA, USA

A close review of the literature about prenatal yoga is highly relevant and important, as research is burgeoning in this field. The goal of this paper is to report on a protocol for conducting a systematic review of experimental studies (RCTs and quasi-experimental studies) that involve prenatal yoga, with attention to the effects on stress, quality of life, mental health, and birth outcomes and attention to the characteristics of the yoga interventions themselves.

 Recommended revisions:

- The Abstract states a need to examine "clinical effectiveness" - however, I am concerned that the state of the current literature would suggest that it will be difficult to do so because not many effectiveness trials have not been conducted at this time. The authors are recommended to review the continuum of efficacy vs effectiveness and clearly delineate whether the systematic review will address clinical effectiveness or efficacy.
- The Abstract does not clearly describe what specific outcomes are of interest for this protocol. It is recommended that the authors clarify the specific aims of the study within the abstract.
- The Aim section should include the specific outcomes of interest - later in the methods section, it is clarified that the researchers intend to focus on primary outcomes of interest (stress, anxiety, depression, and quality of life) and secondary outcomes of interest (birth outcomes - labor duration, pain management in labor, and mode of delivery). These should be clearly stated in the Aims section. It is also unclear what specific physiologic measures will be considered as measures of stress.
- The references do not represent a recent review of the literature, given that the majority of literature cited is more than five years old. For example, the authors are encouraged to consider recent reviews about yoga for various relevant populations conducted by the following teams: Cramer *et al.* ^1^; Ng *et al.* ^2^; Kinser *et al.* ^3^; Smith *et al.* ^4^, among others.

I confirm that I have read this submission and believe that I have an appropriate level of expertise to confirm that it is of an acceptable scientific standard, however I have significant reservations, as outlined above.

## References

[1] : Adverse effects of yoga: a national cross-sectional survey. *BMC Complement Altern Med*.2019;19(1) : 10.1186/s12906-019-2612-7 190 10.1186/s12906-019-2612-7 31357980PMC6664709

[2] : A meta-analysis of the effectiveness of yoga-based interventions for maternal depression during pregnancy. *Complement Ther Clin Pract*.2019;34: 10.1016/j.ctcp.2018.10.016 8-12 10.1016/j.ctcp.2018.10.016 30712750

[3] : Physical Activity and Yoga-Based Approaches for Pregnancy-Related Low Back and Pelvic Pain. *J Obstet Gynecol Neonatal Nurs*.46(3) : 10.1016/j.jogn.2016.12.006 334-346 10.1016/j.jogn.2016.12.006 28302455

[4] : The effect of complementary medicines and therapies on maternal anxiety and depression in pregnancy: A systematic review and meta-analysis. *Journal of Affective Disorders*.2019;245: 10.1016/j.jad.2018.11.054 428-439 10.1016/j.jad.2018.11.054 30423471

[5] : Effects of exercise on pregnant women's quality of life: A systematic review. *Eur J Obstet Gynecol Reprod Biol*.2019;242: 10.1016/j.ejogrb.2019.03.009 170-177 10.1016/j.ejogrb.2019.03.009 30992151

[6] : Non-pharmacological interventions to reduce the symptoms of mild to moderate anxiety in pregnant women. A systematic review and narrative synthesis of women's views on the acceptability of and satisfaction with interventions. *Arch Womens Ment Health*.2019; 10.1007/s00737-018-0936-9 10.1007/s00737-018-0936-9 30613846PMC6987064

[7] : Injuries and other adverse events associated with yoga practice: A systematic review of epidemiological studies. *J Sci Med Sport*.2018;21(2) : 10.1016/j.jsams.2017.08.026 147-154 10.1016/j.jsams.2017.08.026 28958637

[8] : A systematic review of yoga for major depressive disorder. *J Affect Disord*.2017;213: 10.1016/j.jad.2017.02.006 70-77 10.1016/j.jad.2017.02.006 28192737

[9] : Yoga in Pregnancy. *Clinical Obstetrics and Gynecology*.2016;59(3) : 10.1097/GRF.0000000000000210 600-612 10.1097/GRF.0000000000000210 27152528

[10] : Effects of yoga intervention during pregnancy: a review for current status. *Am J Perinatol*.2015;32(6) : 10.1055/s-0034-1396701 503-14 10.1055/s-0034-1396701 25535930

[11] : Yoga for prenatal depression: a systematic review and meta-analysis. *BMC Psychiatry*.2015;15: 10.1186/s12888-015-0393-1 14 10.1186/s12888-015-0393-1 25652267PMC4323231

[12] : A comprehensive review on scientific evidence-based effects (including adverse effects) of yoga for normal and high-risk pregnancy-related health problems. *J Bodyw Mov Ther*.2019;23(4) : 10.1016/j.jbmt.2019.03.005 721-727 10.1016/j.jbmt.2019.03.005 31733753

# Response

**Published:** 2020-01-07

**Authors:** Lisa Corrigan

**Affiliations:** Trinity College Dublin, Ireland

Dear Patricia

Thank you for your review and helpful comments and suggestions.  Your input is valuable to improving our protocol and indeed our systematic review.  We provide a response to each of your suggested revisions:

- The Abstract states a need to examine "clinical effectiveness" - however, I am concerned that the state of the current literature would suggest that it will be difficult to do so because not many effectiveness trials have not been conducted at this time. The authors are recommended to review the continuum of efficacy vs effectiveness and clearly delineate whether the systematic review will address clinical effectiveness or efficacy.

The protocol now reflects your concern and the full review will indeed consider the continuum of efficacy vs effectiveness.  For the purpose of the protocol it was considered important to use the language that is cited in the literature of RCTs and quasi-experimental studies of pregnancy yoga which appears to be predominantly ‘effect’.  While some consider efficacy, it is felt that this continuum of efficacy vs effectiveness will indeed as you suggest form part of the full review where studies will be categorised as being ‘efficacy or effectiveness’ studies.   

- The Abstract does not clearly describe what specific outcomes are of interest for this protocol. It is recommended that the authors clarify the specific aims of the study within the abstract.

The protocol now reflects your concern and outcomes of interest are clearly identified in the abstract thank you for highlighting this.    

- The Aim section should include the specific outcomes of interest - later in the methods section, it is clarified that the researchers intend to focus on primary outcomes of interest (stress, anxiety, depression, and quality of life) and secondary outcomes of interest (birth outcomes - labor duration, pain management in labor, and mode of delivery). These should be clearly stated in the Aims section. It is also unclear what specific physiologic measures will be considered as measures of stress.

The aims section has been amended to include the outcomes of interest.  Stress will include measures of stress by salivary cortisol and/or self-report measures/questionnaires. 

- The references do not represent a recent review of the literature, given that the majority of literature cited is more than five years old. For example, the authors are encouraged to consider recent reviews about yoga for various relevant populations conducted by the following teams: Cramer *et al.* ^1^; Ng *et al.* ^2^; Kinser *et al.* ^3^; Smith *et al.* ^4^, among others.

Thank you for these citations, many of which will form part of the broader discussion in our final review and will contribute greatly to creating a context for our findings.  As this is a protocol to guide how the study will be conducted and structured, only selected references were cited.
